# Collectanea

**Published:** 1831-09

**Authors:** 


### COLLECTANEA.

Floriferis ut apes in saltibus omnia libant,
Omnia nos, itidem, depascimur aurea dicta.

### ANATOMY.

*Nerves of the Cornea.* Professor Schlemm, of Berlin, is said to have discovered these nerves, and that they originate from the superficial branches of the ciliary nerves, and may be traced along the sclerotic coat, and over the orbiculus ciliaris towards the cornea, between which and the sclerotic they penetrate and become imperceptible.

*Anatomical Phenomenon.* M. Combetti, in the sitting of the 23d of May, read a memoir, containing the particulars of the case of a young girl, aged ten, who had recently died in the hospital for Orphans.

Alexandrine Labrosse was born at Versailles in 1821; her father was healthy, but her mother weak, and worn out by excesses of every description. The child came into the world meagre, but well formed; it was very weak, and at two years old had not cut its first teeth. It was not able to articulate a word until after it was three years old, and could not stand alone until it had completed its fifth year. To this backwardness of corporeal developement was added a great imbecility of mind. At nine years old she was admitted into the Orphan hospital, at which time she was labouring under a paralysis of the abdominal extremities. M. Combetti did not see her until January 1831, when she had been three months in bed. Her face was pale, and her features emaciated and oppressed with stupor; she never spoke, and, when addressed, replied in monosyllables, but always to the purpose; she lay constantly on her back, keeping her head inclined towards the left side; she could scarcely move her legs, but they retained all their sensibility; her hands were unaffected. She had long had glandular swellings of the neck; she afterwards had a mild carbuncle on the buttock, and an ulceration of the foot. She was ultimately attacked by an intestinal affection, which carried her off on the 25th of March last. The body was dissected thirty hours after the death. The lungs were found *crepitans*, but full of miliary tubercles. The intestinal surfaces offered no appearance beyond what was usual in cases of similar disease. The cranium was of the ordinary thickness; the meninges offered nothing particular; the brain appeared in its proper state, except that it was rather large. A small sanguineous effusion of recent date was observed in the thickness of the left posterior lobe. The tentorium of the cerebellum being opened, the marrow cut in the direction of the occipital orifice, and the encephalic mass removed and turned over, there was observed, 1, a large quantity of serosity filling the occipital fossa; 2, in the place of the cerebellum, a cellular, gelatinous, semi-circular membrane of about an inch and three quarters diameter transversely, and connected with the medulla oblongata by two gelatinous processes. Near these peduncles were two small white isolated masses about the size of a pea, upon one of which was one of the nerves of the fourth pair: the quadrigeminal tubercles were uninjured; 3, no appearance of the fourth ventricle; 4, the pons varolii entirely wanting, without any appearance of deperdition of substance; the anterior pyramids terminated forkwise by the cerebral peduncle. It appears that the unhappy child had, from her earliest infancy, contracted habits of the most vicious self-indulgence; and M. Combetti, in arguing on the foregoing facts, is disposed to attribute the absence of the cerebellum and pons varolii to a gradual destruction of those parts from disease, and not to any inherent defect of organization. At any rate, the case presents the extraordinary fact of the child having lived for some time in the possession of all its faculties, and even of a certain degree of intellect, though deprived of cerebellum, posterior peduncle, and cerebral protuberance. MM. Geoffroy St. Hilaire, Blainville, Magendie, Flourens, and Serres have been appointed to examine and report on the case.—*Journ. of Royal Institution.*

---

### PATHOLOGY.

THE CHOLERA. *An Experiment proposed, by which the Controversy respecting the possibility of the Importation of the Cholera Morbus by Merchandise, may be finally decided*

It is stated in the Lancet, that M. CHERVIN, the able, and we sincerely believe very honest, opponent of those who contend that the yellow fever is a contagious disease, has recently addressed a letter to the French minister, in which he proposes an experiment that could not fail to determine the very important question, whether cholera can, or cannot, be transmitted by various kinds of merchandize. He recommends that a spot should be selected, in one of the least populous districts of the north-west of France, and that a swift-sailing steam-vessel should be procured to carry from the Baltic a cargo of the foul linen, garments, beds, &c., just taken from the dead and dying of the existing pestilence; that from sixty to one hundred volunteers, of different ages, and temperaments, and habits, (of whom he demands to be the first,) be there clad in these clothes, lodged in the beds, and in every manner exposed to the supposed fomites of the contagion. He thus conceives that this great mercantile and sanatory question may be completely solved. If the one hundred individuals thus situated escape unharmed, restrictions are nugatory, and are prejudicial to the first interests of nations. If, on the other hand, the disease explode amongst the experimentalists, uncertainty will be at an end, precautions must be redoubled, and the philanthropic merchant will learn to submit with resignation to the restrictions imposed on commerce, for the safety of his fellow men.

---

*Lacteal Infection.* M. Guyon recently communicated to the Academy of Sciences of Paris the death of an infant and a dog, who had partaken of the milk of a woman suffering from fever. The former died in thirty hours, and the latter in less than four, exhibiting all the usual symptoms of death from poison.

---

*Bloody Sweat, occurring during the Hysteric Paroxysm.* M. Chaufford relates, in the November number of the Transactions Medicales, a curious case of a girl, aged twenty-one, small, sanguineous, menstruating irregularly, brain but little developed, mind weak, idle and obstinate, addicted to contemplation, and who was persecuted by her parents for having abjured her religion. She fled her paternal mansion, and after seeking various asylums, was admitted into a hospital. She was at that time subject to hysteric attacks, manifested by general convulsions, exquisite sensibility of the pubic and hypogastric regions, &c. When the attack was violent, and continued for twenty-four or thirty-six hours, the patient went into a sort of ecstacy, characterized by fixed eyes, loss of intelligence, &c., and a bloody sweat was poured out by the cheeks and epigastrium; the blood was in small drops, and stained the linen. The whole cutaneous system was injected at the part which was the seat of the hemorrhage, and the skin of this part was of a bright red, and covered with a vascular network. This phenomenon occurred whenever the hysteric catalepsy continued for a long time, or was exasperated by the impatience of the patient. These symptoms continued for three months, and then yielded to revulsive bleedings, and revulsive topical applications.

---

*A remarkable Case of Coryza Phlegmatica.* By the Cavaliere Luigi Sementini, of Naples, M.R.I. &c.

The following account of an uncommon disease, which has recently come under my observation and care, may not be unacceptable. A similar affection has been called by Sauvages and Borsieri, Coryza Phlegmatica or Phlegmatoragia.

The patient, who was about fifty years old, abounding in humours, and of a sanguine temperament, after a strong fit of sneezing, was suddenly affected with a constant running from the right nostril of a clear liquid, in large drops, to the number of twenty-five in a minute, and of twelve at the least; but the diminished number occurred only during the hour after dinner. The discharge increased to the larger number of twenty-five drops per minute in the evening, and continued without interruption during the whole course of the night. Hence the patient was obliged to take his rest in an uneasy position, with his head inclined forward, without which precaution he would have been suffocated. In walking, on the contrary, he went with his head erect, in order that the liquid might fall into the fauces, from whence he discharged it by large mouthfuls, thus avoiding the disgust of having his face and clothes continually soiled.

The fluid in question had a strong saline taste, and the patient was obliged to muffle up his nose during meals, in order that it might not mix with his food. Nevertheless, he was not afflicted with headach, nor with any uneasy sensation in the fore part of the head, nor indeed with any of the ordinary phenomena accompanying such diseases. He only lost a little flesh, and in other respects enjoyed perfect health.

This disease, which, in my time at least, has not been known to occur in Naples, is described by the celebrated Morgagni in his fourteenth Medico-Anatomical Letter, where he treats of the diseases of the head. But the case he describes of a Venetian lady, who was affected with it, and cured by him, differs from that which came under my observation, in that the quantity of the fluid discharged was not above one half, and that it continued for near a year, while in my patient it continued four months; and because the health of the lady was so sensibly affected by it as to endanger her life.

The disease was for a long time treated by me in the accustomed derivative method of foot-baths, blisters, sudorifics, purgatives, &c., but in vain. Afterwards, remembering that the afflicted person was subject to the gout, though in an anomalous and irregular form, I was of opinion that a portion of the gouty humour might have affected the olfactory nerves; and the result of my experience being that no means were more effective than James's antimonial powders to reduce this disease to a state of regularity, and therefore probably to remove this humour from the unaccustomed seat it occupied, I subjected the patient to a course of that medicine. At the end of four days, his head, which had always been free from pain, became slightly affected; and at length, after profuse perspirations, the disease, which had come suddenly, ceased in a manner quite as sudden.

The fluid discharged, although transparent and limpid when it first issued from the nostril, soon became turbid, and acquired a light yellow colour, depositing a flocculent animal substance. In its composition, beside the muriate and phosphate of soda, I found a considerable quantity of urate of soda, especially in the flocculent deposit: there was scarcely any free soda.—*Journal of Royal Institution.*

---

PRACTICAL MEDICINE.

*On the Use of Chlorate of Lime in purulent Inflammation of the Eyes.* By Dr. HERZBERG. (*Journal Complémentaire.*)

M. VOULEZ, of Brussels, has tried this remedy with great success, in all kinds of purulent ophthalmia: he employs it in the following form: Distilled water ℥iv.; Liquor of Labarraque gtts. xv.; three or four drops of this lotion are dropped into the eye four times a day. Dr. HERZBERG has also frequently adopted a similar practice with advantage, and the following cases are given to shew its efficacy.

CASE I. A man, four days after being attacked with gonorrhœa, washed his eyes with his urine, for the purpose of relieving a weakness of his sight, which had resulted from chronic ophthalmia; in a few hours he felt a troublesome sensation in his eyes, which gradually increased to positive pain. To relieve this symptom, he repeated the "urine lotion." At the expiration of a quarter of an hour, the pain increased in severity; the eyes became red, and he could not bear the light. The patient remained in this state twenty-four hours without any assistance. When Dr. H. was consulted, he found the upper eyelids œdematous, and hanging over the lower, which were equally tumefied; there was also a free dicharge of unhealthy-looking pus from the eyes; the conjunctive membrane was turgid, and of a deep red colour; the right eye was most severely affected; pulse hard, full, and quick; occasional shiverings. From the urethra, a discharge of matter similar in appearance to that from the eyes: but little pain in making water. Twelve ounces of blood were taken from the arm, and four doses of calomel, two grains each, were prescribed to be taken each day. Every ten minutes the eyes were injected and bathed with a solution of half a drachm of chlorate of lime in six ounces of distilled water. In less than two hours the pus discharged assumed a more healthy appearance. On the second day the discharge was much diminished, and thinner; conjunctiva less turgid. As there was still considerable pain in the right eye, twelve leeches were applied.

Third day: symptoms alleviated; the cornea could now be seen; it appeared softened, and was covered with several ulcers. To the collyrium before used, half a drachm of tincture of opium was now added. The discharge from the eyes decreased daily; the tumefaction of the conjunctiva diminished; the cornea was brighter and firmer; and the ulcerations decreased in size. Calomel omitted, as symptoms of salivation appeared; the lotion was continued for fifteen days.

Case II. A child, two years of age, was attacked with frequent sneezing and redness of the eyes, with discharge of tears. The following day, the eyelids were swollen and œdematous. The fourth day, Dr. H. found there was an abundant discharge, and the eyelids were tumefied to the size of nuts. Two leeches were applied below each eye, and a grain of calomel was given three times a day. A lotion, composed of a scruple of chlorate of lime in six ounces of distilled water, was instantly applied. The matter discharged soon assumed a more healthy appearance, and was much diminished in quantity in twenty-four hours. The calomel and chlorate lotion were abandoned on the eighth day, and in about a fortnight the cure was completed by occasionally dropping into the eyes a little tincture of opium.

Case III. A child was attacked with ophthalmia four days after birth: the disease was neglected for six weeks. Upon examination, several ulcers were detected in the right eye; the conjunctiva of a deep red colour, and tumefied; copious discharge of thick unhealthy-looking pus. The chlorate of lime lotion was alone employed, and in eight days the cure was effected. No internal remedies were used. To hasten the healing of the ulcerations on the cornea, a little laudanum was now and then instilled into the eyes.

Case IV. A female infant was attacked with ophthalmia three days after birth. For two months no other "remedy" was applied than a lotion consisting of the urine of *all the members* of the family. The mother washed her face with a sponge that had been used to remove the discharge from the eyes of the infant, and in twenty-four hours she was also attacked with inflammation of the left eye. Dr. H. was now consulted. The child was cured in ten days by the lotion before mentioned. Local bleeding and calomel were thought necessary for the mother; and she also used as a lotion a solution of two drachms of chlorate of lime in six ounces of water: her cure was effected as speedily as that of her infant.

---

*Cure of Fever by Powder of Holly-leaves.* On the 11th of April, M. Rousseau announced to the Academy of Sciences of Paris, that in three distinct cases of recent occurrence, fever had been completely cured by a few doses, of a drachm each, of the powder of holly-leaves, diluted with half a glass of water. The Academy directed the Medical Committee of the Prix Monthyon to take cognizance of the cases.

On the 23d of May, M. Deleschamps, a young chemist, announced that he had succeeded in obtaining a new vegetable matter from the bark of holly, to which he had given the name of ilicine, and which may be substituted for quinia in the treatment of intermittent fevers. It will be recollected than an extract of the bark of willow, called salicine, has already been suggested as a substitute for sulphate of quinia: should experience prove that the qualities of these two matters are at all comparable to those of quinia, their low price will render the discoveries highly important to the lower classes.—*Journal of the Royal Institution.*

---

*Preservative against Smallpox and Measles.* On the 13th of June a letter was read from M. Remy, a physician at Chatillon, detailing some experiments which he had recently made on chloride of lime as a preservative against the smallpox. During the last autumn he had observed that, out of some hundred individuals whom he had vaccinated, nearly five sixths had not taken the infection properly; whereas in the spring, although he had used matter precisely similar, every case succeeded. He then recollected, that during the autumn he had constantly carried in his waistcoat pocket a small packet of chloride of lime, and felt convinced that the non-reception of the virus must have been occasioned by that circumstance. He therefore, in a village where the smallpox was raging with such violence that there only remained twelve individuals subject to the infection who had not been attacked, caused those twelve to be washed twice a week with a solution of chloride of lime, and gave them at the same time two drops of the solution in a glass of *eau sucrèe.* Two of them had a slight eruption, similar to a vaccine which has not taken well; the other ten remained constantly with patients suffering from the smallpox, without the least symptoms of illness. In another village where the smallpox also raged, there were fifteen individuals liable to take it; ten of them were subjected to a similar treatment, and wholly escaped the malady; two of the other five caught the complaint. The same treatment was tried on four individuals under the influence of the smallpox; the result was an increase of inflammatory symptoms, which were removed by bleeding; the progress of the eruption appeared arrested, the pustules remained in the same state as when first washed with the solution, and then dried away very slowly. This letter was referred to MM. Magendie and Serres. On the 20th of June, M. Chevalier reminded the Academy that he was the first who had suggested the use of chloride of lime as a preservative against the smallpox, long before the experiments of M. Remy. He also stated that the chloride of lime might likewise be used as a protection against the measles, by keeping in the chamber of the child, whom it was desired to preserve from infection, a saucer of dry chloride of lime, renewed from time to time, and dipping its shirts in a solution of one ounce of concentrated liquid chloride in twelve quarts of water. —*Ibid.*

*On the Pathology and Treatment of Pertussis.* By W. R. Whatton, Esq., Surgeon, Manchester.

I venture, without apology, to forward the following observations on pertussis. The disease has visited us in Manchester and the surrounding neighbourhood, very severely, in the form of an epidemic, during the last three months, and the mortality has been correspondingly extensive. At such periods, when cases of any particular disease are frequent, valuable opportunities are afforded for its investigation, for the examination of remedies and their effects, and for necroscopic observations; and, as it behoves the practitioner to acquire his knowledge on any and every occasion, how and when he may, such opportunities ought not to be neglected.

An excellent paper on this subject, by Mr. Sandwith, of Bridlington, printed in the third number of the North of England Medical and Surgical Journal, has induced me to transmit these remarks, partly in corroboration of his judicious observations, and partly with the view of submitting my own opinions and experience to the test of examination.

By following the doctrines of Watt, and some other subsequent writers, who have lately, and so ably, inculcated the dependence of pertussis on bronchial inflammation, instead of classing it among nervous disorders, I am desirous of aiding in the endeavour to rout the abettors of the Cullenian opinions of its purely spasmodic nature, and of offering my feeble support to the new theory; convinced as I am, most forcibly, both of the excellence of its foundation, and of its consistence with every sound principle of physiological and pathological reasoning. The idea that "the proximate or immediate cause of pertussis seems to be a viscid matter, or phlegm, lodged upon the bronchia, trachea, and fauces, which sticks so close as to be expectorated with the greatest difficulty, and by its nature excites spasmodic coughing," ought long since to have been exploded and forgotten; and "the irritability of the stomach and mucous glands," so fondly advocated and acted upon by the spasmodic pathologists, will, I hope, be exchanged for the better notions of inflammation, irritation, and morbid secretion.

The writings of Dr. Watt, Mr. Alcock, Dr. Hastings, and others, demonstrate, incontestably, the highly inflammatory appearances of the mucous membrane of the bronchial tubes, after death, in fatal cases of pertussis, and my own dissections have always tended to corroborate their remarks. The membrane of the glottis and larynx, and of the trachea and divarications of the bronchi, is generally found highly inflamed, exceedingly vascular, thickened, and of a dull-red colour; frequently coated with frothy puriform secretion, in such quantities as to impede entirely the passage of air, and occasionally studded with ulcerations.

I have observed, however, that the substance of the lungs has, in some instances, been more affected than the bronchial tubes themselves; and in one case particularly, along with a large abscess in the right lobe, there was an extensive collection of matter situated between the pleura costalis and the ribs on the left side. This child was of a delicate strumous habit, and had been much debilitated by long previous illness. It is clear, I think, that the delicate mucous membrane of the glottis and cartilages of the larynx is, in the first instance, affected by the inflammation, and that it becomes afterwards the fixed seat of the disease. We may easily conceive that these parts would be quickly thrown into spasmodic action, and the membrane being highly vascular, in a short time the mucous fluid, which it separates from the blood for the purpose of moistening the air passages and organs of the voice, would be acted upon by the inflammation, and be so thickened and inspissated, as to become a frequent source of uneasiness and difficult expectoration to the patient.

The degree of febrile excitement is as yet moderate; the respiration is not much oppressed; the pulse, however, is quick, and we have torpid bowels and scanty and high-coloured urine, followed by hoarseness, cough, and redness of the eyes; and this may be considered, in general, the common order in which the symptoms announce themselves. As long as the disorder continues in the subacute stage, or, in other words, as long as the inflammation does not extend itself beyond the glottis and larynx, the case is favorable.

But the whole extent of the tracheal membrane is extremely liable to a continuation of the inflammation, which was at first seated in the glottis and larynx only; and in an exact ratio as it descends will be the violence of the spasm, the rapid succession of the strokes of the cough, the loudness and sharp tone of the hoop, and the difficulty and distress in recovering the inspiration of air. There are also an accompanying dryness and stiffness of the membrane lining the fauces and larynx, which are productive of heat and continual thirst to the little patient, and which add greatly to the general amount of uneasiness. The pulse too is exceedingly quick, wiry, and hurried. During the actual existence of the fits, the functions of the lungs are entirely suspended, and consequently the arterialisation of the blood cannot proceed; great determination to the head takes place, and the countenance becomes livid and dark, the lips blue, and there is excessive agitation over the whole frame, until a sufficient quantity of air is recovered to relieve the asphyxia and to balance the circulation. By the time the fit is nearly over, a small quantity of tough knotty mucus is brought up, sometimes streaked with blood, and this is usually lodged upon the surface and sides of the tongue, but from the anxiety of the child to inspire, it is generally got out of the way by swallowing, and is seldom or very rarely thrown off. When this severe process is gone through, and the child is somewhat calm again, a free burst of perspiration usually shews itself over the forehead and lips, the infant recovers to a certain extent, and lies down till he is again disturbed by the approach of another attack. If the case has been very urgent, and the child has suffered severely during the fit from the exhaustion and fatigue, he lies quite passive and still, and will not allow any one to interfere with him, or even to speak, so great is his horror in anticipating the period of coughing, and so earnest his desire of delaying to the latest moment the return of the paroxysm. Should the disease be allowed to continue in this state without relief, the interval between the fits is gradually contracted, the difficulty of breathing is increased, the lividity of the countenance becomes permanent, and the child either dies from the consequences of the inflammation, from excessive exhaustion, or goes off suddenly in convulsions, from the undue pressure and effects of highly-carbonized blood on the brain.

The chief aim, in the treatment of the subacute, or milder cases, of pertussis seems to be to put the disease into a safe train, and to conduct it through its course by the exhibition of such remedies as are calculated to reduce the inflammation, and thereby to lessen the irritation, to promote an easy and free expectoration, and to keep the circulation upon the surface, but, above all, to prevent its assumption of the graver form. The bowels should be well and effectually opened with calomel and jalap powder, and leeches should be applied over the cartilages of the larynx, so as to remove the congestion, or to the back of the neck, or between the shoulders, if there be also much determination upwards. These may be repeated from time to time, until the breathing be effectually relieved. A mixture composed of four grains of tartrate of antimony dissolved in six ounces of water, and sweetened with some simple syrup, is what I usually employ, regulating the dose according to the age and strength of the child, and repeating it often enough to procure vomiting after the fits of coughing. Pediluvium, and a grain or two of James's powder, at bedtime. When the disease assumes the more acute form, energetic measures are demanded to subdue the inflammation. In some of these the pulse arrives at an almost countless celerity, the dyspnœa is continuous between the fits of coughing, and the child has every symptom of approaching suffocation. Unless prompt and decisive relief is given here, the case very soon gets beyond our reach, collapse follows, and the patient sinks.

From a child of four or five years of age, I have usually taken three or four ounces of blood from the arm, or jugular vein, from a free opening, with the happiest and most decided effects, and have occasionally found it requisite to repeat the bleeding in four or six hours, if the pulse rise to any thing near its original fulness, and do not immediately become soft and compressible. Even under these circumstances, it is generally indispensable to have recourse to the application of leeches to the thyroid cartilage from time to time, and to exhibit freely the antimonial mixture. A small blister should also be applied over the chest.

The following case, occurring in one of my own children, will be sufficient, as an example of the treatment necessary for subduing this formidable disease in its more urgent form:

A. B. W., a healthy boy, aged four years, of a sanguine temperament, was affected with pertussis about the middle of April. He was visited by a professional friend, and was treated with antimonials in the usual way, had leeches occasionally to the throat, and the bowels were regulated by proper aperient medicines. The case was mild, and rather favorable for the first six weeks, but became gradually more urgent, when he was removed into the country for the additional benefit of change of air. On the 6th of June, at one o'clock in the morning, he was suddenly seized with convulsive cough, excessive dyspnœa, and lividity of countenance; pulse 160, wiry, and hard, with that indescribable recoil which is so truly indicative of a high degree of inflammatory action. Being, fortunately, on the spot, I immediately drew blood from the arm until the pulse fell to 120, and was much softer, and the respiration had become greatly relieved. The blood was highly cupped and buffed. In the space of four hours, however, the pulse had nearly reached its former height, and the dyspnœa had again become exceedingly urgent. Three ounces more blood were drawn from the opposite arm, with a correspondent beneficial effect; the pulse fell to 100, and again became soft, and comparative ease in the breathing followed immediately. An equable perspiration broke out over the whole surface, and the boy slept comfortably for two hours.

On the 7th two leeches were applied to the integuments over the larynx, and a powder, consisting of two grains of calomel and ten of powdered jalap, given; four loose motions were obtained from the medicine, and the child appeared in every respect improved, though pale and very feeble; pulse 100, soft, and free from recoil; cough much reduced.

8. Some lingering difficulty of breathing yet remaining, three leeches were again applied to the throat, and a powder given every four hours, containing two grains of James's powder. Cough still less.

9. The aperient powder repeated, two leeches again applied to the larynx; breathing good, and the cough easy, and the intervals between the fits much lengthened.

10. Pulse 100; convalescent.

In a week the child was able to go out into the air, and has continued quite well ever since, perfectly free from cough, and his appetite excellent.

The only remarks I have to add to the foregoing observations are, that I am averse to the use of the warm bath in urgent cases of pertussis in very young children. In no one instance has the immersion failed to induce great excitement, to accelerate the pulse, and to increase the uneasiness and difficulty of breathing. It is necessary, also, in ordering the application of leeches to the larynx, to have them fixed over the cartilages, and not on the soft parts on either side. I have in several cases experienced the greatest difficulty in arresting the flow of blood from the thyroid veins, after the use of leeches in these situations, where the cough has been violent; the nitrate of silver frequently fails, and one is obliged to sit by the patient, making pressure with the finger for several hours before the hemorrhage will yield. I know instances where, from a very trifling neglect, the child has bled to death.—*Lancet.*

---

*Ergot of Rye.* We perceive, by the number of the Revue Medicale for July 1830, that, at the session of the Royal Academy of Medicine at Paris, of the 8th of June preceding, M. Gerardin stated, that "he was in the United States at the period when ergot was in its greatest vogue as a means of accelerating the delivery of the fœtus, but so many instances of still-born children occurred, that the government had instituted an inquiry into the causes by which these were occasioned: with the result of this inquiry, he added, he was unacquainted, it not having been published at the period of his departure for France."

On this subject M. Gerardin has, from some cause, been undoubtedly led into an error. The government of this country has never, we believe, directed any inquiry to be made of the nature alluded to; nor are we aware that, taking the whole United States, the proportion of still-born children is greater here than what occurs in Europe. Some inquiry of the kind may. however, have been instituted by one of the State Medical Societies.

With respect to the ergot, the general opinion of the profession in this country is still favorable. That there do occur many cases in which the article may be employed by the judicious practitioner to accelerate labour, with decided advantage to both mother and child, there can be no possible doubt; but from what we know of the very hasty and indiscreet manner in which it is too often resorted to, we fear that its introduction into obsterical practice has, upon the whole, been productive of more harm than good.—*North American Med. and Surg. Journal.*

---

### SURGERY.

*Lithotrity.* In our number for June last, we mentioned the report that M. Civiale had lately submitted to the French Academy of the great success of numerous cases of lithotrity, in most of which he had operated himself. The subject is too important, in every point of view, to be thus briefly dismissed, especially as the medical committee who were appointed to investigate the subject have thrown some doubts upon the statement given by M. Civiale of the favorable results of the lithotritic operation. The substance of M. Civiale's report is given in the number of our Journal we have just referred to, and we find, from the Archives Gènèrales, Juin 1831, p. 276, that, in answer to this report, Messrs. Larrey and Boyer have recently addressed the Academy. Having investigated the cases that had occurred at the Hôpital Necker, of which an account had been given to the Academy by M. Civiale, they state that M. Civiale appears to have been desirous of making prominent the advantages only of lithotrity; and that, apparently for the purpose of depreciating the operation of lithotomy, "to the profit of lithotrity," he had passed over in silence the want of success which ought to have been attributed to the latter operation. Messrs. Larrey and Boyer further state, that, from the information they obtained, it appears that the number of patients who died after the lithotritic operation was quite as numerous as those upon whom lithotomy had been performed in other hospitals. We cannot but sincerely regret such perplexing and conflicting statements upon so important a subject. How is it possible, with such contradictory evidence, to form any opinion of the claims which lithotrity really possesses to the notice of surgeons? M. Civiale will, of course, make some reply to the counter-statement of Messrs. Larrey and Boyer; and we shall keep a close eye upon the French Journals, in order that we may furnish our readers with any further information that may transpire upon the subject. As the case stands at present it is interesting, not merely from its professional importance, but because it involves the accuracy of some of the parties, all of whom we are inclined to regard as "honourable men."

---

### *Use of the Nitrate of Silver Ointment in Chronic Conjunctival Inflammation, and in the early and latter Stages of Gonorrhœa.* By Hugh Neill, Esq., M.R.C.S. Ed.

It was in October 1827 that I first had an opportunity of seeing this ointment introduced to practice; I was then for some months a visiter at the Royal Westminster Ophthalmic Institution, and each day a number of cases were brought before my view, when its beneficial effects were so developed, that my attention was drawn towards it in the strongest manner.

The ointment, for the efficacy of which I shall vouch, is composed of ten grains of nitrate of silver, finely levigated, and a drachm of simple unguent, well mixed together. I started when I heard of its component parts, and saw Mr. Guthrie, the first who used it, fearlessly apply it between the lids, and rub it over the eyeball. Still more astonished was I when, a few days after, the beneficial effects of its action upon the diseased surface were developed. From this time forward it was used in almost all cases of chronic conjunctival inflammation, with a success which I may call universal. It was used in many instances of the purulent ophthalmia of infants, and in each case in which I saw it applied, the best results followed.

Between the disease of mucous membranes there is a close resemblance, and it was from seeing the case which I shall now mention that I was first struck with the propriety of adopting a treatment, which for nearly four years I have followed without the slightest cause for regret. A case presented itself in the institution, of conjunctival inflammation; the accustomed treatment afforded no relief, and the man had been for a considerable time under the medical officer's hands, when it was discovered that there was gonorrhœal discharge from the urethra. Mr. Guthrie recommended the introduction of a bougie armed for about an inch with the ointment. The result was favorable; the running in a few days was checked, and the disease of the conjunctiva then readily yielded to the treatment which it had before rejected.

In very many of our European climates I have had opportunities of trying the bougie thus armed, and I can decidedly pronounce, that in the primary stage of gonorrhœa, its introduction has, in the milder cases, in a few hours checked the complaint; in those that were more acute, it has paved the way for a rapid cure.

In the north of Ireland, 1829, a boy was brought to me who had a most hideous eversion of the upper and under lids of the right eye, caused by two fungous growths from diseased conjunctiva. I immediately used applications of solid nitrate of silver. These, with steaming over warm water, were followed up until the excrescences had been reduced from the size of large beans to the level of the healthy surface of the lids. The ointment I then used for a short time, and a disease existent for more than a year was expelled in seven weeks.

When up the Mediterranean in 1828, as surgeon of the steam ship Duke of York (now H.M.S. Messanger), our firemen were sadly annoyed by a sulphurous effluvia from coal procured at Savona in Italy, and their eyes assumed that bleared appearance which may be seen in almost every smoky Irish hut. Steaming over warm water, and one application of the ointment, always gave relief.

A man, a free liver, and formerly a prize-fighter, called upon me about ten weeks since; he had been for some years nearly blind. The shadow of light was all that was admitted, owing to a network of blood-vessels thrown across the front of the eyes, the meshes of which were so closely interwoven, that hardly a ray could gain admission to the retina. I tried the use of the ointment, and although most of the vessels of the sclerotical conjunctiva contracted and regained their natural appearance, yet the vessels of the conjunctiva of the cornea were nearly *in statu quo.* I scarified, but it was of no avail; I then removed a portion of their trunks with scissors, applying to the bleeding ends of the vessels the solid nitras argent. I now am applying the ointment: the cornea is rapidly regaining its transparency, and the patient has vision, which for nearly seven years was lost.

To speak again of gonorrhœa. I have had many cases of it up to twelve months standing, and it is seldom that, even at that late stage, my friends have to ask for a third "poke" of the bougie. If the trial and its results should prove as beneficial in other hands as it has done in mine, I shall be glad; for this simplicity of treatment in the early and latter stages of gonorrhœa will conduce much to the alleviation of a most teasing, and, to both patient and surgeon, tedious disease and irksome attendance.—*Lancet.*

---

*Fracture of the Cranium, accompanied with Loss of Brain and Hernia Cerebri; successfully treated.* By Joseph K. Swift, M.D. of Easton, Penns.

On the 16th of December, 1825, I was called to Jacob Sherrer, of this neighbourhood, who was thrown from his horse while in a state of partial intoxication, although his general habits were those of sobriety and industry. He had fallen against the sharp angle of a large stone, and received a fracture of the parietal bone, commencing about its middle, and extending downwards to the squamous suture, upwards to the sagittal suture, and backwards to the os occipitis, or very near to it. The wound of the scalp was between six and seven inches in length. About two square inches of the parietal bone were comminuted and driven through the dura mater into the substance of the brain; some of the fragments of bone were visible; but most of them were hidden in the brain, being forced edgewise into it; one piece in particular, nearly an inch in diameter, was covered to half an inch in depth, and so completely concealed, that I did not discover it until all the other fragments had been removed. I placed the pieces together, and finding that they would not fit, concluded that there must be a portion remaining, and found it with a probe. All the pieces so removed were entirely separated from the integuments.

Three other fragments, equal to two square inches, near the occipital bone, were turned at a right angle, directly into the substance of the brain, but these were not entirely separated from the scalp, although the dura mater and other membranes were lacerated. I elevated these portions of bone to their natural level.

A surface of brain was thus exposed, from three to four inches square, in several places penetrated by fragments of bone to the depth of more than an inch, denuded completely, and lacerated by fragments of various sizes.

After carefully removing these fragments, I separated nearly a tablespoonful of brain, which was lying loose upon the wounded surface; besides which, a small quantity of brain was observed upon the stone where the accident occurred, but how much I did not ascertain.

A spicula of bone had penetrated the longitudinal sinus, from which a considerable quantity of blood had escaped previous to my arrival; it was then readily commanded by a dossil of lint. The left side was paralyzed, the injury being on the right.

I drew the integuments together, and confined them by a couple of stitches, one at the upper, and another at the lower, angle of the wound, leaving a disunited space in the middle of about two inches. A light flaxseed poultice was directed to be placed over the wound; the patient's head to be kept greatly elevated, and cold applications to be applied, excepting to the part occupied by the poultice. I bled him three pints, gave a dose of cream of tartar and jalap. Diet: bread and water. Room to be kept dark, and absolute quiet enjoined.

During the bleeding he vomited freely and fainted twice. Notwithstanding the extent of injury which the brain had sustained, he retained almost entire possession of his senses; his feelings were excited, and his memory somewhat affected, with an increased disposition to talk, but volition was not impaired. During the time I was operating on and dressing his head, he answered all questions in a rational manner, knew by the sensation when I removed a piece of bone, and conversed much with his wife and children.

On the following morning the dressings were removed; numerous portions of brain adhered to them, perhaps a teaspoonful. A serous fluid was discharging. Bled him three pints.

In the evening, discharge of serum, mixed with blood. Bled him one quart.

About the third day from the receipt of the injury, a portion of brain, equal in size to a common walnut, protruded from the integuments, by which it was considerably constricted at its base. Good pus was discharged in small quantity, and the tumor sloughed off in the course of three or four days, and was succeeded by two or three others of smaller size, which underwent the same process. For several subsequent weeks there was a copious secretion of healthy pus; and to be brief, the patient recovered without a single unpleasant symptom, to the utter astonishment of myself and of all who saw him.

The indications I pursued throughout this case were, the most rigid adherence to the antiphlogistic regimen; an upright position of the head; the constant use of applications; absolute rest and quiet, and a careful preclusion of every exciting cause. Venesection was pushed to such an extent as to keep the pulse constantly below the fever point: whenever it evinced an increase of action I bled him, mostly *ad deliquium*: the bowels were slightly constipated, rendering purgatives occasionally necessary.

The paralysis had much abated before the cicatrization of the wound, and the slight disturbance of his mental faculties also subsided.

Nine months have now elapsed since the occurrence of the accident, and the patient, a farmer, is able to attend his business. His left side is still weaker than his right, although its use is completely restored. In August last, I called him into my office to shew his head to my friend Mr. Franklin Peale, and in answer to the many inquiries we made him, he stated that he thought his faculties better than they were before his injury; that he could calculate with facility, and that previously to the accident, he was subject to a very severe periodical headach, to which he is now a stranger.

The motion of the brain is distinctly visible through the integuments, and the vacuity from loss of brain and bone would contain rather more than a half a gill of fluid.

May not the extent of laceration of the dura mater have been favorable to the fortunate issue of the case, by allowing a free discharge of pus and extravasated fluids, and consequently removing all pressure from the brain? At all events the case decidedly proves that injuries of the brain, with loss of its substance, and consequent laceration of the membranes, are not necessarily mortal.

March 31, 1826. The patient continues well, and experiences little or no difficulty in attending to his ordinary duties. The left arm is still not so strong as the right, and its motion not quite so free, but it is progressively recovering.

My friend Dr. Cooper, of Easton, attended the patient with me after the second or third day of the accident.

*Note*, by Samuel George Morton, m.d., one of the physicians to the Philadelphia Almshouse Infirmary:

"Happening to be at Easton during the present month, (July 1830,) Dr. Swift politely took me to see the individual whose case is recorded in the preceding communication. Four years and a half have now elapsed since the accident occurred, and the subject of it remains precisely in the situation described by Dr. Swift: he assured me of his increased facility in making calculations, and of the entire absence of headach, which had greatly afflicted him prior to the receipt of the injury. He is of a robust frame, and can at present endure as much hardship as at any former period of his life. No paralytic symptoms remain."

The cavity in his head remains as great as ever, but the integuments have become much thickened, and the pulsation of the brain, though obscure, is still evident to the touch.—*N. American Med. and Surg. Journal.*

---

*Section of the Sciatic Nerve.* This operation has been performed by Dr. Malagodi, of Bologna, for the cure of an obstinate neuralgia, seated in the superficial nervous ramifications of the leg and foot, of eleven years' duration, and which had resisted a multitude of remedies. Previous to the operation, Dr. M. tried a number of experiments upon dogs, from which it appeared that the section of the sciatic nerve, two thirds down the thigh, produces paralysis, and subsequently atrophy, but not gangrene. That this paralysis extends from the middle of the leg to the extremities of the toes; that the leg does not, however, lose the power of sustaining the body, or become unfit for the purposes of locomotion, the muscles which move it not being paralysed, and the knee-joint not being affected.

It is also proved by amputations of the thigh, that the division of the nerve does not compromise life. These results determined Dr. M. to perform the operation, which he accordingly did on the 5th of March, 1828 in the presence of Profs. Matheo Venturoli, Paolo Baroni, and M. Joseph Bertolotti. The patient was a man aged thirty-one years. The incision was made about three inches above the popliteal hollow, and was two inches and a half in extent. At the moment when the section of the nerve at the upper part of the wound was made, there occurred a trembling of all the limbs, and a pain similar to the pricking of needles rapidly extended from the place of section to the spinal column and brain, and the patient fainted. When he recovered, which was very soon, Dr. M. requested him to be very attentive to his feelings, and the nerve was then divided at the lower portion of the wound, without the patient being aware of it. Upwards of two inches of the nerve were thus removed in order to prevent its reunion. From the moment of the division of the nerve, all pain ceased, and had not returned five months after, when Dr. M. communicated the case to the Medico-Chirurgical Society of Bologna. There was paralysis, however, of the leg and foot, with tingling and sensation of weight in these parts, but there remained an obtuse sensibility of the inside of the leg.—*Gaz. Méd.* and *American Journal of Med. Sciences.*

---

*Strangulated Hernia, promptly reduced by the Application of Belladonna Ointment.* An old man had had, for twelve years, an inguinal hernia of the right side, which had always been reduced without difficulty. In stepping into bed one night, the hernia passed down: he was immediately attacked with colic, vomiting, &c. The tumor was of a red colour. The taxis was tried in vain: an ointment, principally composed of belladonna, was then applied. The pain was quickly relieved; in an hour the tension of the tumor had disappeared, and a third application of the ointment was followed by reduction of the rupture.—*Ibid.*

---

### MATERIA MEDICA.

*The Ointment of Mezereon as a Dressing to maintain a Permanent Discharge from Issues, &c.* It is well known that in many cases, where the Unguent. Cantharid. is employed as a dressing to a denuded surface, strangury is developed, and occasions the patient much distress. To obviate this inconvenience, Professor Hufeland has proposed to substitute the Unguent. Mezerei, which he thinks possesses many advantages over the other. The following is the formula which he recommends for its preparation: ℞. Extract spirituous, Cort. Mezerei, ʒj.; Axung. porc. ℥ix.; Ceræ. alb. ℥j.; salve extr. in unc. und alcoholis, adde axungiam et ceram et misce modico calore continue agitando, usque ad perfectam evaporationem alcoholis: tunc cole.—*Journal der Practischen Heilkunde*, band. 70, stuck. 1, and *American Journ. of Med. Sciences.*

---

*New Styptic.* M. Bonafous has communicated to the Royal Academy of Medicine, that he has succeeded, with a powder composed of equal parts of rosin, carbon, and gum arabic, in arresting hemorrhage from large arteries. The author related several cases in which its application to the divided brachial artery in man, to leech bites, to the carotid artery of a horse, &c. had entirely arrested the flow of blood.—*Gazette Medicale*.

---

### MEDICAL JURISPRUDENCE.

*Poisoning by the Topical Application of Arsenic.* The danger of applying arsenic to large surfaces of the skin has long been known to medical men: the article is, however, frequently resorted to by irregular practitioners, and not unfrequently with fatal effects. In the Transactions Médicales, we find related the cases of three children who were destroyed by the use of this poison. These three children, who were of the ages of seven, nine, and eleven, had scald head, to which the mother, at the recommendation of a charlatan, applied an ointment, which he assured her would cure her children in a few days. In a few hours the children were attacked with pains all over their body, dreadful colic, and the next day the youngest died, and the day after the two others.

Dr. Fristo, of Sierck, examined the bodies of these children, and the ointment which had been applied to their heads. The bodies were found swelled and ecchymosed; the membranes of the brain greatly injected; the substance of this organ inflamed, presenting a number of sanguinolent spots; lungs hepatized and engorged; stomach phlogosed; ecchymoses in the duodenum and small intestines.

The ointment was found to contain arsenic.—*American Journ. of Med. Sciences.*

---

